# Analysis of Audiometric Differences of Patients with and without Tinnitus in a Large Clinical Database

**DOI:** 10.3389/fneur.2017.00031

**Published:** 2017-02-09

**Authors:** Dominik Gollnast, Konstantin Tziridis, Patrick Krauss, Achim Schilling, Ulrich Hoppe, Holger Schulze

**Affiliations:** ^1^Experimental Otolaryngology, Department of Otorhinolaryngology, Head and Neck Surgery, Friedrich Alexander University Erlangen-Nürnberg (FAU), Erlangen, Germany; ^2^Department of Physics, Center for Medical Physics and Technology, Biophysics Group, Friedrich-Alexander University Erlangen-Nürnberg (FAU), Erlangen, Germany; ^3^Audiology, Department of Otorhinolaryngology, Head and Neck Surgery, Friedrich Alexander University Erlangen-Nürnberg (FAU), Erlangen, Germany

**Keywords:** human, pure tone audiometry, speech audiometry, sensorineural hearing loss, conductive hearing loss, tinnitus model

## Abstract

Human hearing loss (HL) and comorbidities like tinnitus pose serious problems for people’s daily life, which in most severe cases may lead to social isolation, depression, and suicide. Here, we investigate the relationship between hearing deficits and tinnitus. To this end, we conducted a retrospective study on anonymized pure tone and speech audiometric data from patients of the ENT hospital Erlangen in which we compare audiometric data between patients with and without tinnitus. Overall data from 37,661 patients with sensorineural (SHL) or conductive HL (CHL) with (T, 9.5%) or without (NT, 90.5%) a tinnitus percept in different age groups and with different tinnitus pitches were included in this study. The results of the pure tone audiometry comparisons showed significant differences in T patients compared to NT patients. In young patients, we generally found lower hearing thresholds in T compared to NT patients. In adult patients, differences were more heterogeneous: hearing thresholds in T patients were lower in low frequency ranges, while they were higher at high frequencies. Furthermore, lower thresholds were more often found in CHL patients and could rarely be detected in SHL patients. In speech audiometry, only CHL patients with high-pitched tinnitus showed lower thresholds compared to NT patients’ thresholds. The results of this study may point to a biologically plausible functional benefit on hearing thresholds in HL tinnitus patients. We hypothesize that the physiological mechanism of stochastic resonance counteracts HL by adding neuronal noise to the system. This neuronal noise may induce changes in the auditory pathway and finally—as a side effect of threshold improvement—lead to the development of a tinnitus percept. We propose a general model of changed hearing thresholds in T patients, being either decreased or increased compared to NT patients.

## Introduction

Hearing is crucial for audio-verbal communication in humans and by that essential for social interactions. Consequently, hearing impairment pose serious problems for the people’s daily life, which in most severe cases may lead to social isolation, depression, and suicide (1, 2). The fact that our western societies face increasing noise exposure in daily routine, work, and spare time adds to the problem and results in continuously increasing numbers of people suffering from hearing impairments. Studies in the United States revealed about 9% of the general population being affected by hearing deficits in 2000 (3)—with an overrepresentation of 15% in children (4)—and an increase of cases over 10 years when assessed with more advanced testing procedures (5).

In general, factors leading to this high prevalence of hearing impairments are diverse. Consequently, the cause for hearing impairment may be located within different anatomical structures. Hearing disorders may have their origin in outer or middle ear, causing conductive hearing loss (CHL), or sensorineural structures (starting in the cochlea and including all structures along the auditory pathway), causing sensorineural hearing impairments. Of course patients can also suffer from a combination of conductive and sensorineural hearing loss (HL), either with unilateral or bilateral impairments. Probably, the most common cause for sensorineural hearing impairments is noise-induced HL (NIHL), which can lead to a number of secondary symptoms like tinnitus [for review, see Ref. (6)], depression (7), or hyperacusis (8). With increasing HL over time, the capability of speech discrimination is affected, generating difficulties in everyday life communication (9). As Cruickshanks and colleagues (10) demonstrated that the risk of HL is increased by almost 90% every 5 years, the demographic changes in most industrialized western countries will make age-dependent HL even more relevant in the future.

Tinnitus is a widespread, but poorly understood symptom often seen in HL patients. The prevalence in the general population is assumed to be at around 10–15% (11). Men seem to be more often affected then women up to the age of 75, when prevalence is about equal for both genders (12). About 1–2% of the tinnitus patients state their quality of life being significantly decreased by their phantom percept (13). Although HL and the prevalence of tinnitus are increasing with age (14), it is still under debate if age-related HL or age-related changes in physiological processes in the central and peripheral auditory system are the source of the increasing prevalence of tinnitus (15).

Population studies revealed that individuals with tinnitus on average suffer from stronger HL in high frequencies; patients with low-pitched tinnitus (below 1,500 Hz) show stronger low frequency HL than patients with middle- or high-pitched tinnitus [e.g., Ref. (16–19)]. In any case, the question remains why in some patients with HL subjective tinnitus is developing at all. In a recent study (20), we put forward a model for the physiological improvement of hearing thresholds, which as side effect also explains the development of tinnitus. The model is based on the idea that the auditory system tries to compensate for a HL by means of stochastic resonance (SR) at the receptor level. SR refers to the phenomenon that weak signals that are sub-threshold for a given sensor still can be detected and transmitted by that sensor if noise (internal or external) is added to the sensor input, both in technical and physiological systems (21–24). We further assume that HL leads to an unequal distribution of spectral input into the auditory system, with a reduced input from the affected spectral ranges. Our model proposes that the impaired hearing thresholds within those frequency channels may be improved again (at least to a certain degree) by means of SR. Obviously, for SR to work, internal noise has to be generated within the auditory system and fed back to the receptor level (20). We propose that this internal noise is reflected in neuronal hyperactivity. If the HL is permanent, the neuronal hyperactivity that enables SR to compensate for increased thresholds may subsequently cause neuronal plasticity along the auditory pathway and finally may lead to the development of a phantom percept, i.e., subjective tinnitus. In that sense, the model views tinnitus as a side effect of a mechanism within the auditory system that seeks to optimize signal transmission at the receptor level. If this model would be true, we would expect that, in tinnitus patients, initial HL should be compensated to a certain degree, resulting in overall better hearing thresholds in tinnitus patients compared to non-tinnitus patients with comparable damage in the auditory system.

In this retrospective study, we search for data in support of this hypothesis by performing a fine-grained analysis of the audiometric data of over 37,000 patients with different forms of HL, and with or without a tinnitus percept.

## Materials and Methods

We performed a retrospective study on anonymized audiometric data from patients with HL who came to the ENT hospital in Erlangen for medical examination. Therefore, no declaration of consent was required by German law. All data were collected between the years 2000 and 2015, HL patients who complained about experiencing pure-tone tinnitus percepts were classified as tinnitus patients (group T) and patients without complains about any form of tinnitus were classified as non-tinnitus patients (group NT). Patients with other forms of tinnitus (e.g., noise-like tinnitus) were not included in this study. Applied audiometric methods were pure-tone and speech audiometry for multisyllabic numbers. Data from 37,661 patients (74,976 ears) including all groups of age [median (25, 75% quantil): 42 (21, 58)] were investigated. Patients were not characterized by their gender or by former or current pathologies not affecting hearing.

### Testing Procedures

Standardized audiometric testing instruments of an audiological clinic were used for this study. All devices fulfilled the necessary requirements according to ISO 8253-1 and 8253-3. The following audiometric methods were performed: pure-tone audiometry: air conduction hearing level thresholds were measured for both ears separately for every patient. Analyzed frequencies were 250; 500; 750; 1,000; 1,500; 2,000; 3,000; 4,000; 6,000; and 8,000 Hz and HL was calculated (range: −10–130 dB). Speech Audiometry: using the Freiburger test, multisyllabic numbers were presented to each ear separately. For these data, acoustic levels at 50% understanding were calculated and used for further analysis (range: 0–120 dB). Audibility was directly linked to the acoustic level (range: 0–100%). The Freiburg multisyllabic numbers were used since it belongs to our standard procedure to determine the level of 50% understanding. This value usually correlates with the hearing threshold in the low frequency region and is less affected by high-frequency HL. We did not use the monosyllabic words since monosyllabic perception is usually measured at higher levels.

Tinnitus characteristics were determined in terms of signal type and signal level measured in decibel HL by comparison of the internal tinnitus with external sound from the audiometer. Three types of signals were possible: broadband noise, narrow band noise (1/3 octave bandwidth), and pure tones between 0.25 and 8 kHz. Tinnitus loudness was determined by increasing the signal level above hearing threshold slowly in steps of 1 dB and asked the subjects for a comparison with their tinnitus percept.

### Data Preprocessing and Statistical Analysis

Data preprocessing was performed with a custom-made Matlab 2008 program (MathWorks, MA, USA). For statistical analysis Statistica 2007 (StatSoft. Inc., OK, USA) was used. Patients were classified within the preprocessing into groups based on their audiometric data: mild to medium symmetric sensorineural HL in adults [SHL, mean air-bone–gap ≤5 dB across all frequencies, HL difference between both ears <20 dB, maximal HL ≤40 dB; number of ears: *n*(T) = 4,390, *n*(NT) = 26,142], symmetric CHL in adults [CHL, mean air-bone gap >5 dB across all frequencies, HL difference between both ears <20 dB, maximal HL ≤40 dB; *n*(T) = 2,538, *n*(NT) = 24,726], and a group of children and adolescents under 18 years with symmetric HL [HL difference between both ears <20 dB, maximal HL ≤40 dB; *n*(T) = 234, *n*(NT) = 16,946] without further subdivision in SHL or CHL patients.

Furthermore, adult subjects were grouped by age: young adults 18–39 years [SHL: *n*(T) = 1,418, *n*(NT) = 8,540; CHL: *n*(T) = 630, *n*(NT) = 7,642], elder adults 40–60 years [SHL: *n*(T) = 2,164; *n*(NT) = 11,236; CHL: *n*(T) = 1,182; *n*(NT) = 9,254], and seniors >60 years [SHL: *n*(T) = 808; *n*(NT) = 6,366; CHL: *n*(T) = 726; *n*(NT) = 7,830]. The subjective pure-tone tinnitus frequencies were grouped by pitch: low-pitched [<1,000 Hz; *n*(SHL) = 507; *n*(CHL) = 387], medium-pitched [1,000–4,000 Hz; *n*(SHL) = 875; *n*(CHL) = 662], and high-pitched [>4,000 Hz; *n*(SHL) = 3,008; *n*(CHL) = 1,489].

In pure tone audiometry, we aimed to quantify the potential threshold decrease or increase tinnitus may have on hearing thresholds in each single tested hearing frequency. To this end, the difference of mean HL in each frequency was calculated for each age and tinnitus pitch group and its age matched NT patients (mean HL difference, where positive values indicate a threshold decrease, negative values indicate an increase of hearing thresholds by tinnitus in decibel) and compared by multifactorial ANOVAs. Generally, for statistical population analysis, parametrical tests like Students *t*-test and multifactorial ANOVAs were used. Tukey *post hoc* tests enabled detailed analysis within the ANOVAs. Additionally, we compared the paired pure tone and speech audiometry data of 2,548 patients in which both audiometric methods were allied by multiple linear regressions and 2-factorial ANOVA (Figure S1 in Supplementary Material).

## Results

### Pure-Tone Audiometry in Patients with and without Tinnitus Percepts

#### Hearing Thresholds of Children and Adolescents

The HL of young patients without (NT) and with reports of tinnitus (T) was compared. To rule out any age bias due to audiometric limitations in infants, we tested first if any difference in hearing thresholds of very young children (aged 1–9 years) and adolescents (aged 10–17 years) could be found. Neither in NT (*t*-test, mean ± SD: children 20.5 ± 17.3 dB, adolescents 19.2 ± 21.6 dB, *p* = 0.15) nor in T patients group (*t*-test: children 16.0 ± 12.6 dB, adolescents 14.3 ± 19.8 dB, *p* = 0.17) any significant differences were found. Therefore, all subjects were pooled and HL was analyzed by a 2-factorial ANOVA with the factors frequency and group.

**Figure 1:**
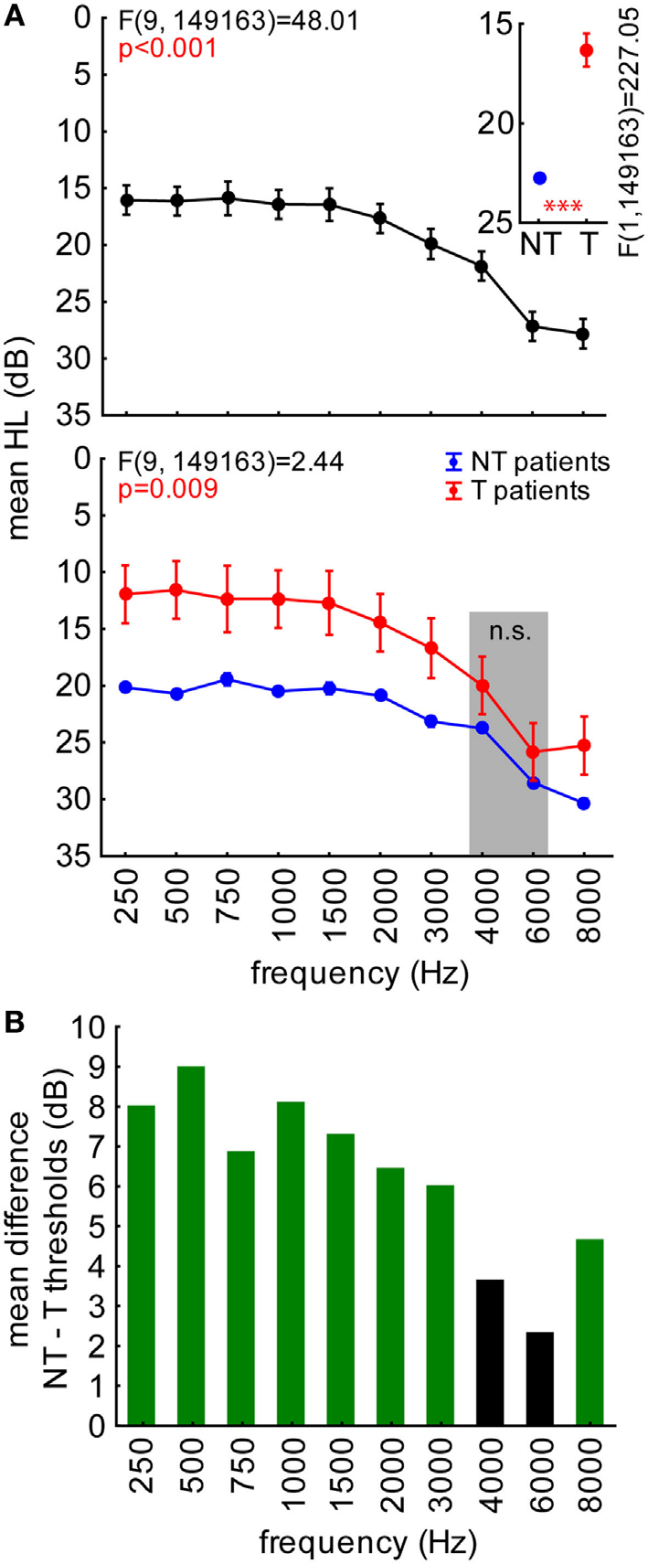
**Hearing loss (HL in decibel) in young patients (aged 1–17 years) with and without tinnitus**. **(A)** Results of a 2-factorial ANOVA on HL with factors frequency (upper panel) and patient group (inset), interaction plot at the bottom. Symbols depict mean values; whiskers give 95% confidence intervals. Asterisks depict significance value of the 1-factorial ANOVA part: ***p < 0.001. Gray-shaded area indicates two frequencies not significantly different between groups (Tukey *post hoc* tests). **(B)** Mean differences between the HL of NT [blue in **(A)**] and T patients [red in **(A)**] quantifying the threshold difference referred to as mean HL difference, where positive values indicate lower, negative values higher hearing thresholds in decibel. Values significantly different from 0 are colored green (single sided *t*-tests).

Figure 1A depicts this variance analysis. The upper panel shows an increase of mean HL toward higher frequencies in all patients (Figure 1A, upper panel). Interestingly, young people with tinnitus generally suffered less from HL than non-tinnitus patients (Figure 1A, inset), overall showing about 5 dB lower mean hearing thresholds. They were especially less affected at frequencies below 4,000 Hz and above 6,000 Hz (Figure 1A, lower panel, Tukey *post hoc* tests, always *p* < 0.05). In Figure 1B, the mean HL difference of NT and T patients is given as a function of frequency. Each green bar indicates the significant difference (tested by single sample *t*-tests) of the HL at a given stimulation frequency. Again, all frequencies except 4 and 6 kHz show significantly positive values indicating lower hearing thresholds in T compared to NT patients.

Note that this young patients group is not representative for patients affected by tinnitus in the “general population” where usually only adults are considered (1, 25). For that reason, further analyses were focused on adult groups with SHL and CHL. In these patients, we were able to analyze a significantly larger number of patients’ ears.

#### Hearing Thresholds in Adults with and without Tinnitus

**Figure 2:**
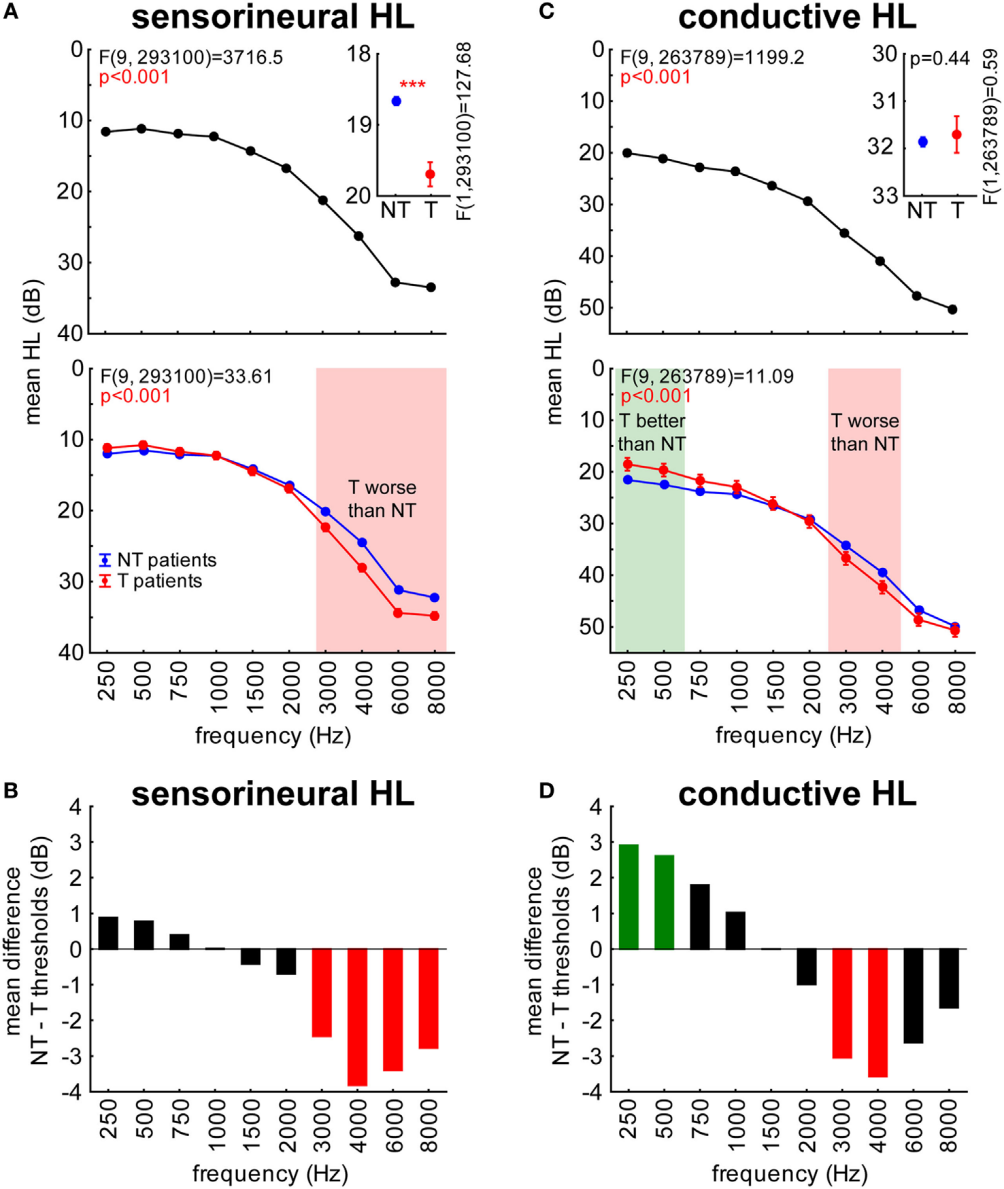
**Hearing loss (HL in decibel) in sensorineural (A,B) and conductive HL (C,D) patients**. Symbols as in Figure 1. **(A,C)** Red areas indicate frequencies where T patients exhibit higher hearing thresholds compared to NT patients, the green area indicates frequencies where T patients show lower thresholds than NT patients (Tukey *post hoc* tests). **(B,D)** Red bars indicate significantly higher thresholds, green bars significantly lower thresholds.

In a first overview, NT and T patients’ audiograms separated for their cause of HL (sensorineural or conductive) were analyzed by 2-factorial ANOVAs with the factors frequency and group as depicted in Figures 2A,C and further illustrated by the HL difference in Figures 2B,D, respectively.

In adult patients, we generally found that T patients’ thresholds in lower frequency ranges were lower compared to NT patients, while in other frequency ranges there was no difference or, especially above 2 kHz, those patients showed higher thresholds compared to NT patients: a detailed analysis revealed that for both groups, SHL (Figure 2A, upper panel) and CHL (Figure 2C, upper panel), HL significantly increased from low to high frequencies. HL in general was higher for CHL patients (30.9 ± 20.0 dB) than for subjects suffering from SHL (18.3 ± 16.7 dB; *t*-test, *p* < 0.001).

In patients with SHL, the analysis revealed the opposite result seen in young patients, namely mean thresholds across all frequencies being higher in patients with tinnitus compared to patients without such a mispercept by roughly 1 dB (Figure 2A, inset). In the interaction of both factors, it became clear (Tukey *post hoc* tests, *p* < 0.001) that T patients only showed significantly higher thresholds compared to NT patients at frequencies above 2 kHz (Figure 2A, lower panel). This result is further illustrated in Figure 2B showing the mean HL difference, which also revealed a significant threshold increase at frequencies above 2 kHz for SHL patients with tinnitus.

In patients with CHL, the analysis of the factor group showed no significant difference between T and NT patients when thresholds were averaged across all frequencies (Figure 2C, inset). Nevertheless, the interaction analysis (Figure 2C, lower panel) revealed significantly lower thresholds in T patients for frequencies below 750 Hz but higher thresholds compared to NT patients in the range of 3–4 kHz (Tukey *post hoc* tests, *p* < 0.05), which was further supported by the HL difference analysis (Figure 2D).

**Figure 3:**
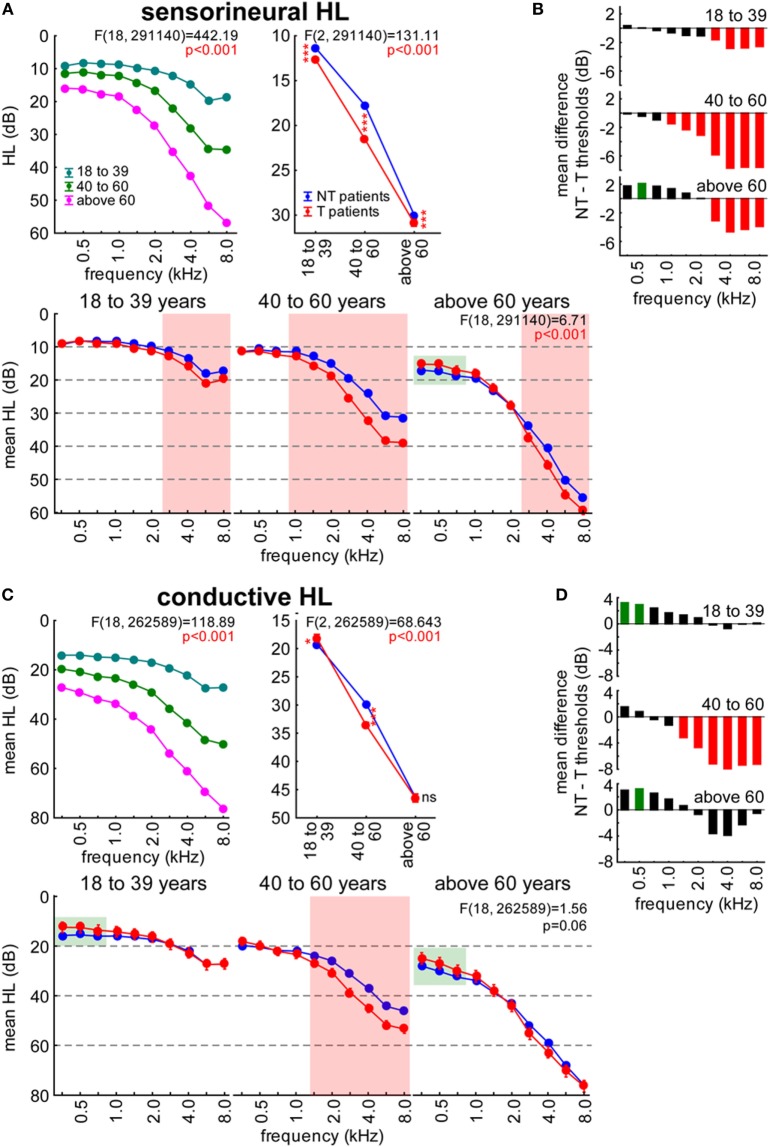
**Age dependency of hearing loss (HL in decibel) in sensorineural (A,B) and conductive HL (C,D) patients**. **(A,C)** show the interaction plots of the 3-factorial ANOVAs of HL with factors age, patient group, and frequency. Asterisks depict significance levels of the Tukey *post hoc* tests in the interaction plot of age X patient group: ns, not significant, *p < 0.05, ***p < 0.001. Red shaded areas indicate frequencies with T patients exhibiting higher hearing thresholds compared to NT patients (Tukey *post hoc* tests separately for each frequency). Green shaded areas indicate frequency ranges (low: <1 kHz, mid: <4 kHz, high: ≥4 kHz) with T patients exhibiting lower hearing thresholds compared to NT patients (2-factorial ANOVAs for frequency ranges and patient groups). **(B,D)** give the mean HL difference of NT and T patients with red bars indicating significantly higher thresholds, green bars significantly lower thresholds in T patients compared to NT patients.

Next, we analyzed different aspects of our patient cohort to outline some additional characteristics of the threshold differences between T and NT patients. First, we focused on the age dependency of thresholds as a function of the existence of a tinnitus percept: SHL patients of all ages showed higher hearing thresholds when affected by tinnitus, while CHL patients with tinnitus only showed such higher thresholds in the age group of 40–60 years. This was assessed by 3-factorial ANOVAs with the factors frequency, age and group as depicted in Figures 3A,C. Lower hearing thresholds in T patients only reached significance when analyzed across frequency ranges (low = < 1 kHz, mid = < 4 kHz, high = ≥ 4 kHz; green shaded areas in Figures 3A,C) but could be further identified when analyzed by HL differences for specific age groups (Figures 3B,D).

Patients with SHL (Figure 3A) replicated the results shown in Figure 2 averaged over all three age groups when factors frequency, group, or the corresponding interaction were analyzed. For the factor age [(F2, 291,140) = 11,515.0, *p* < 0.001], we found lower HL in 18- to 39-year-old adults (12.0 ± 0.2 dB) compared to 40- to 60-year-old adults (19.6 ± 0.15 dB) and adults above 60 years (30.4 ± 0.2 dB). The interaction of age and frequency is given in the upper left panel of Figure 3A and the interaction of age and group in the right upper panel. There, T patients showed generally higher thresholds than NT patients (Tukey *post hoc* tests), which is carved out in the three-way interaction in the lower part of Figure 3A: in SHL, Tinnitus patients’ higher frequencies showed significantly larger thresholds than those of non-tinnitus patients (cf. red shaded areas; statistical tests were performed separately for every stimulation frequency). When 2-factorial ANOVAs [factors frequency range (low, mid, high) and group] were performed, significantly lower hearing thresholds in T patients could be detected for low frequencies in the above 60 years group only (green shaded area in Figure 3A). The HL difference (Figure 3B) for the three different age groups showed corresponding frequency ranges affected, with higher values at high frequency ranges of the tinnitus patients and lower values at 750 Hz in the above 60 years tinnitus patients.

In CHL patients (Figure 3C), we found a similar age-dependent HL as above [F(2, 262,589) = 5,794.5, *p* < 0.001; 18- to 39-year-old adults: 18.7 ± 0.15 dB, 40- to 60-year-old adults: 31.7 ± 0.3 dB, adults above 60 years: 46.5 ± 0.4 dB]. Again, a significant interaction of age and frequency as well as age and group was found (Figure 3C, upper panels). Across all frequencies, CHL T patients’ hearing thresholds differed by age, as we found higher threshold in 40- to 60-year-old adults but lower threshold in 18- to 39-year-old adults. No overall significant differences were seen in adults above 60 years (Tukey *post hoc* tests; Figure 3C, upper right panel). In the three-way interaction (Figure 3C, lower panel) higher thresholds in T patients were found at frequencies above 1 kHz in 40- to 60-year-old adults (cf. red shaded area, statistical tests were performed separately for every stimulation frequency), while significantly lower hearing thresholds in T patients could be detected at low frequencies in 18- to 39-year-old adults and the adults above 60 years of age only [2-factorial ANOVAs for frequency ranges (low, mid, and high) and group, green shaded area in Figure 3C]. Correspondingly, in the analyses of HL differences (Figure 3D) of CHL patients, we found significantly lower values for low frequency thresholds in 18- to 39-year-old adults and adults above 60 years of age with tinnitus while 40- to 60-year-old adults with tinnitus showed significantly higher values in the mid- to high-frequency range.

**Figure 4:**
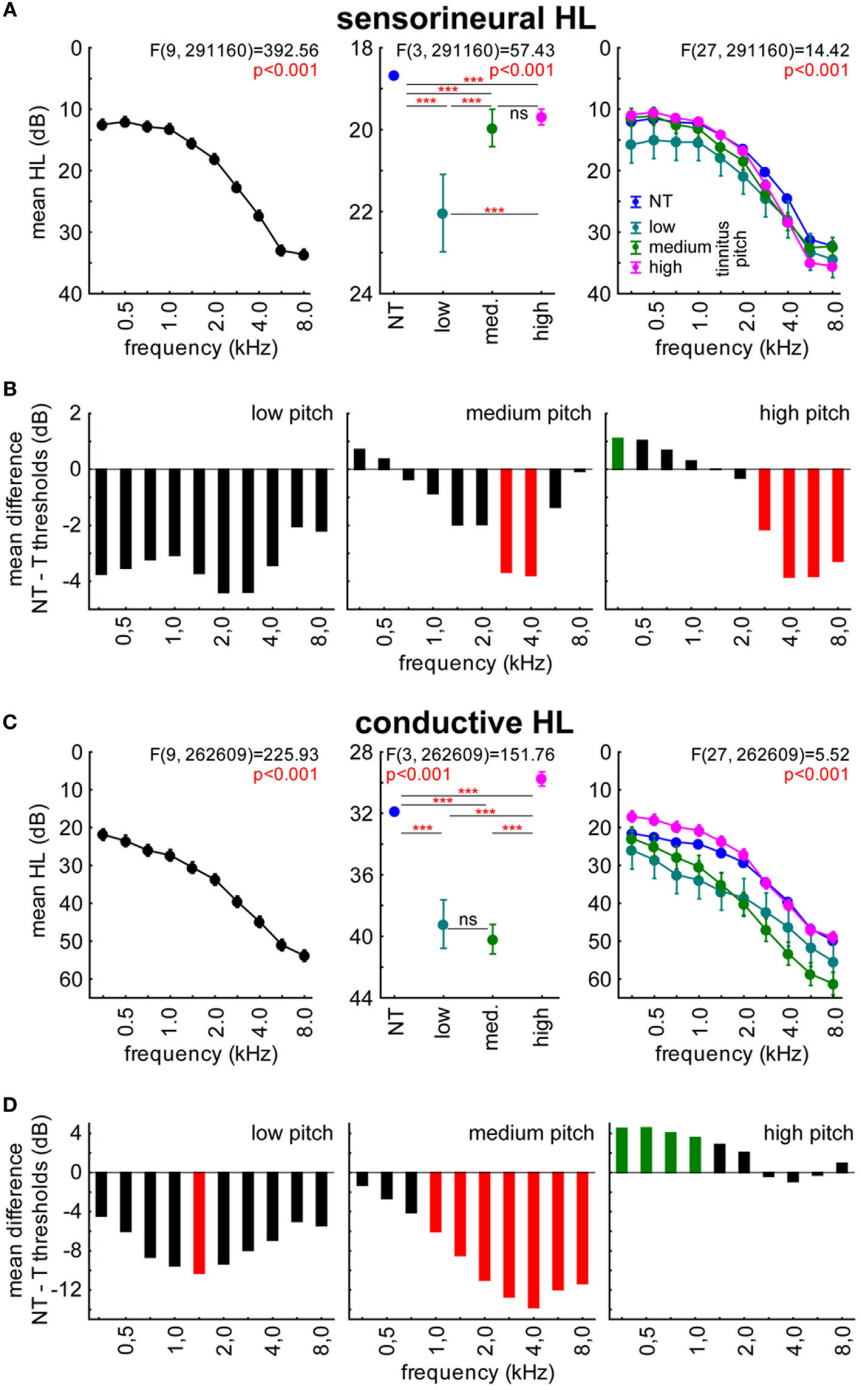
**Relation between hearing loss in (HL in decibel) and tinnitus pitch in sensorineural (A,B) and conductive HL (C,D) patients**. **(A,C)** show the results of the 2-factorial ANOVAs of HL with factors frequency and perceived tinnitus pitch (including NT patients) in the two patient groups. Asterisks depict significance levels of the Tukey *post hoc* tests: ns, not significant; ***p < 0.001. **(B,D)** The threshold difference between T and NT patients is given, with red bars indicating significantly higher thresholds, green bars significantly lower thresholds in T patients compared to NT patients.

From Figures 2 and 3, it became clear that potential effects of the perception of tinnitus on hearing thresholds follow a frequency-specific pattern: in T compared to NT patients, lower thresholds were only observed in the low-frequency range while higher thresholds were exclusively seen in the high-frequency range. As pitches of tonal tinnitus percepts most frequently are located in the high-frequency range, we hypothesized that the higher hearing thresholds in T patients may be due to masking of the perception of tones in that frequency range. To test this hypothesis, we analyzed if there is a relation between the perceived tinnitus pitch and the hearing thresholds of patients by 2-factorial ANOVAs with the factors frequency and perceived tinnitus pitch (NT, low, medium, and high tinnitus pitch). The results of these analyses are depicted in Figure 4. We found higher thresholds in SHL T patients rather being independent of tinnitus pitch. Lower threshold in CHL T patients on the other hand were most prominent in patients with high-pitched tinnitus percepts.

In detail, in SHL patients’ data (Figure 4A, center panel), we found higher thresholds in T compared to the NT patients being most prominent but frequency unspecific in low-pitch tinnitus patients (Tukey *post hoc* tests), but also significant in the medium and high-pitch tinnitus patients, especially for frequencies above 2 kHz (Figure 4B). In CHL patients, a somewhat different picture emerged (Figure 4C). While low- and medium-pitch tinnitus patients showed generally higher thresholds than NT patients when averaged across all stimulation frequencies, the high-pitch tinnitus patients did show lower thresholds compared to the NT patients’ thresholds (Figure 4C, center panel). This difference (Figure 4D) was most prominent for frequencies below 1,500 Hz in high-pitched T groups, while the higher values in tinnitus patients with medium-pitched tinnitus frequencies started at 1,500 Hz and reached up to the highest tested frequency of 8 kHz (Tukey *post hoc* tests). For low-pitched T patients, thresholds were again generally higher compared to NT patients.

In summary, the results of the pure tone audiometry comparisons showed significant differences in hearing thresholds of T compared to NT patients in both directions. In young patients with tinnitus, we generally found significantly lower thresholds almost independent of stimulation frequency (Figure 1) or perceived tinnitus pitch (not shown). In adult patients (Figures 2–4), data were more heterogeneous: in T compared to NT patients, lower hearing thresholds were found at low frequencies only, while higher thresholds were generally found at high frequencies. Furthermore, lower thresholds in T patients were more often found in CHL patients and could rarely be detected in SHL patients.

### Differences in Speech Audiometry between Patients with and without Tinnitus

**Figure 5:**
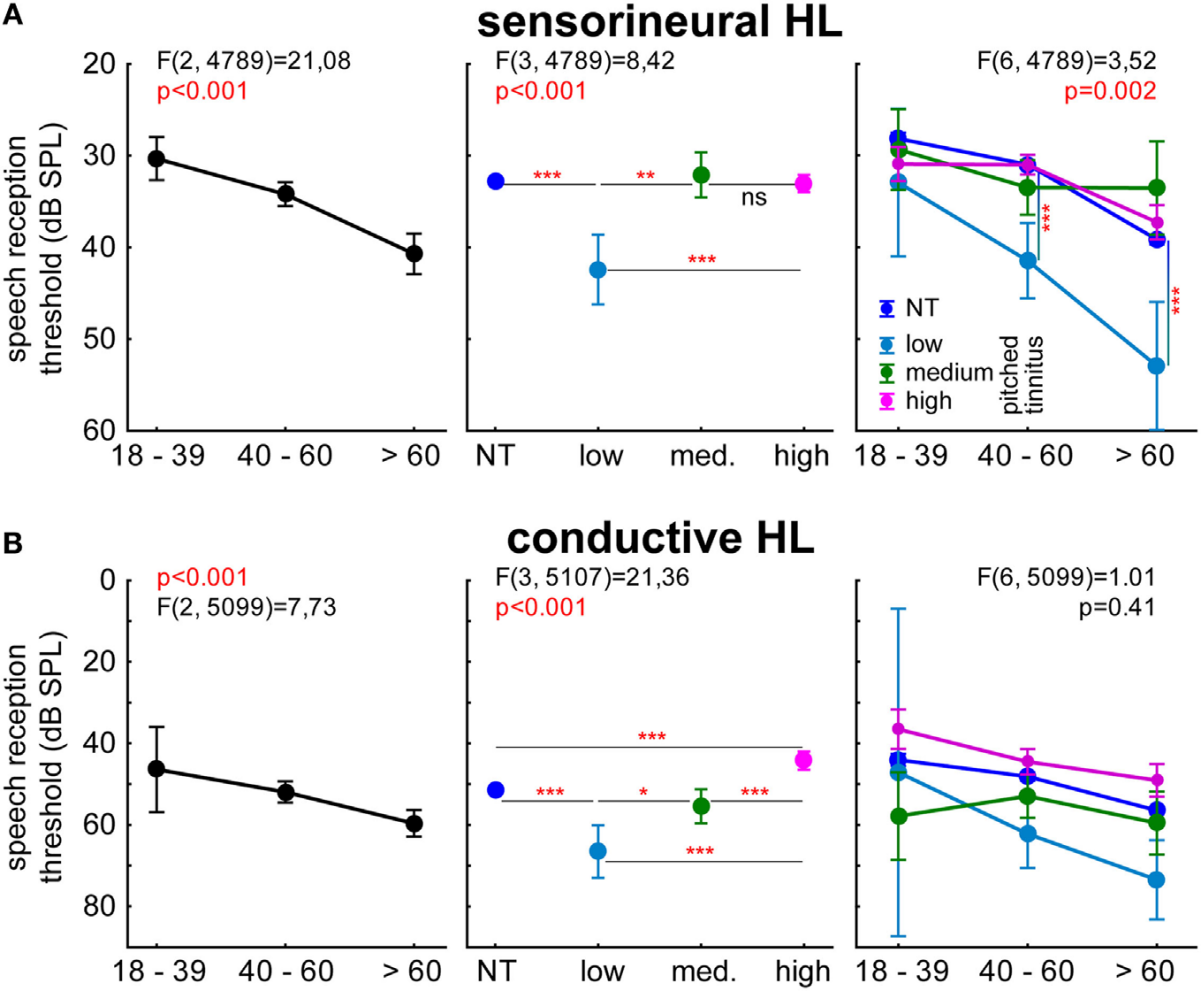
**Speech reception thresholds (decibel SPL) of sensorineural (A) and conductive HL (B) patients in the Freiburger speech intelligibility test (multisyllable numbers)**. 2-factorial ANOVAs of the 50% threshold with the factors age and perceived tinnitus pitch (including NT patients). Asterisks depict significance levels of the Tukey *post hoc* tests: *p < 0.05, *p < 0.01, ***p < 0.001.

In order to examine the potential differences in speech comprehension between both patient groups, we analyzed the results of the Freiburger test, which examines the intelligibility of multisyllabic numbers. Figure 5 shows the 2-factorial ANOVA of the speech reception thresholds with factors age group and tinnitus pitch group, tested for the two major patients groups separately.

**Figure 6:**
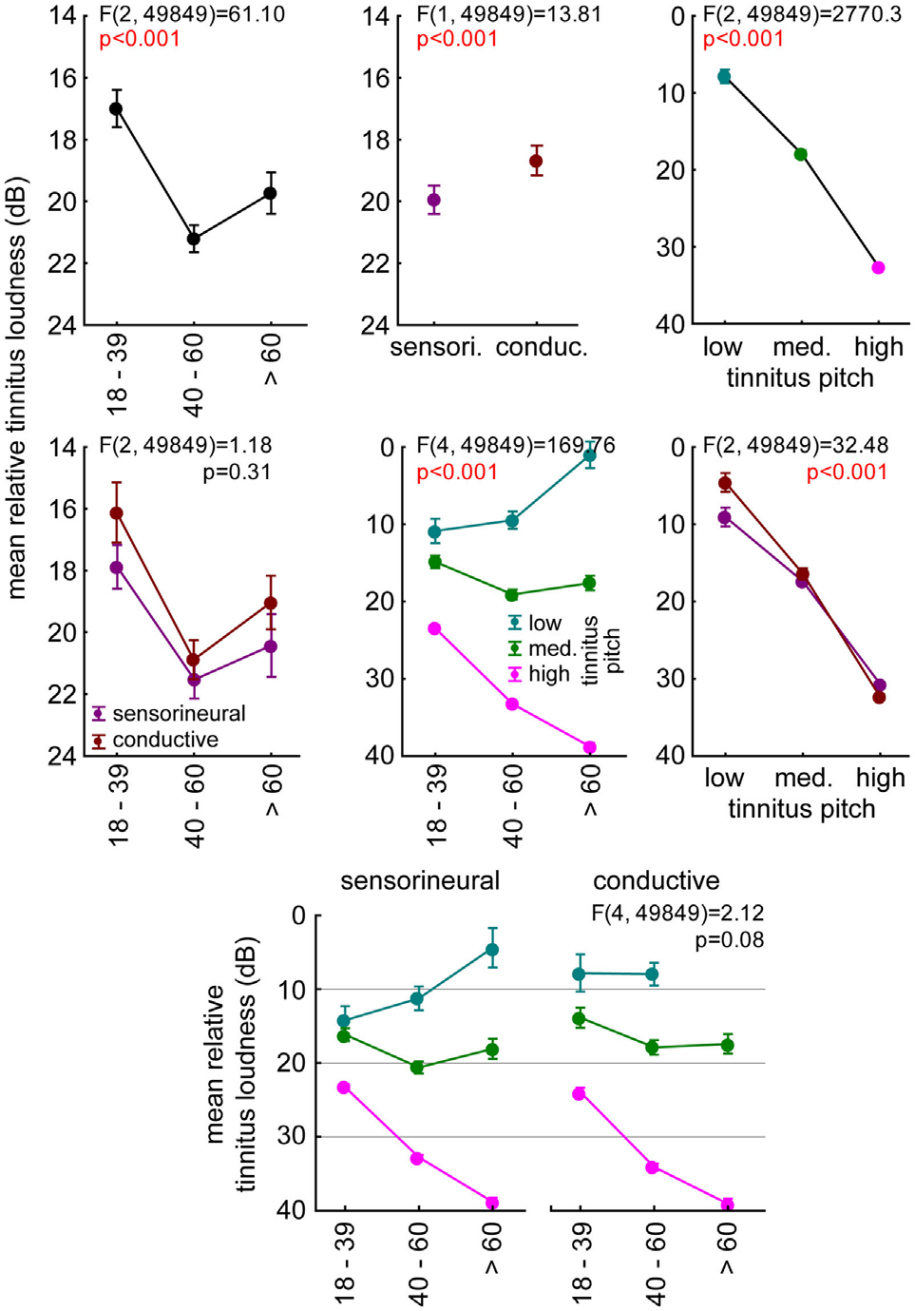
**Relation between mean relative tinnitus loudness (dB) and age, tinnitus pitch, and hearing loss (HL)**. The individual tinnitus loudness was calculated relative to the threshold at that frequency. Results of the 3-factorial ANOVA of tinnitus loudness relative to hearing threshold with the three factors described above. The upper three panels represent the 1-factorial part of the ANOVA results (age, HL, tinnitus pitch), the center line panels show the 2-factorial ANOVA interaction plots (age vs. HL, age vs. tinnitus pitch, tinnitus pitch vs. age), and the bottom line panel shows the interaction plot of all three factors.

Variance analysis of speech audiometry in SHL patients (Figure 5A) showed the expected dependency of speech reception thresholds on age (left panel), but in addition, a stronger impairment of speech intelligibility in patients with low tinnitus pitch in comparison to all other groups (Tukey *post hoc* tests), the medium and high tinnitus frequency perceivers were not significantly different in their understanding of numbers compared to each other and to the NT patients. We did find a significant interaction of both factors (age group and tinnitus pitch), with Tukey *post hoc* tests revealing stronger impairments in adults above 39 years of age and with tinnitus pitches below 1 kHz (Figure 6, right panel).

In CHL patients (Figure 5B), we found a comparable age dependency of the speech reception thresholds (Figure 5B, left panel) as in SHL patients as well as a decrease of speech intelligibility in low-pitch tinnitus patients. In contrast to SHL patients, CHL high-pitched tinnitus patients showed an increase of speech intelligibility thresholds of 7 dB compared to NT patients’ thresholds (Figure 5B, middle panel). No interaction of both factors was found, indicating a threshold decrease in high-pitched tinnitus patients at all ages (Figure 5B, right panel).

To control for any direct effect of the individual hearing threshold on speech reception threshold, we analyzed the relation of both thresholds by multiple linear regressions, center of gravity comparisons, and a 2-factorial ANOVA of the individual threshold differences of the two, pooled across all patients. The results of these analyses are depicted in Figure S1 in Supplementary Material and show no qualitative differences between the age or tinnitus groups in the first two analyses. Nevertheless, the differences between low and medium-pitched tinnitus patients and high-pitched tinnitus and NT patients emerge in the 2-factorial ANOVA and further support the finding, that low/medium pitch tinnitus patients show a different pattern of threshold loss compared to high-pitched tinnitus and NT patients, namely a lower distance between speech reception and pure tone thresholds.

### Analysis of Tinnitus Loudness

The subjective tinnitus loudness perceived by the patients of the two groups relative to their hearing thresholds turned out to be not a good predictor of speech intelligibility. An analysis of this relative tinnitus loudness by a 3-factorial ANOVA with the factors age group, HL group, and tinnitus pitch is given in Figure 6. The tinnitus perception was loudest in 40- to 60-year-old adults (Figure 6, upper left panel, Tukey *post hoc* tests, always *p* < 0.001) and in the SHL patients (Figure 6, upper center panel), particularly in the high-frequency pitched pure tone tinnitus percepts (Figure 6, upper right panel, Tukey *post hoc* tests, always *p* < 0.001). No interaction of age and HL group indicated a parallel shift of tinnitus loudness (Figure 6, center left panel). Furthermore, we found significant interactions of age and pitch (Figure 6, center panel) with a decrease of tinnitus loudness with age for low frequencies and increase for high frequencies. We also found a significant interaction of pitch and HL group (Figure 6, center right panel) with louder low-pitched tinnitus percepts in SHL patients (tukey *post hoc* test, *p* < 0.001) and louder high-pitched tinnitus in CHL patients (tukey *post hoc* test, *p* < 0.01). Most interestingly, the lack of a significant three-way interaction (Figure 6, bottom panel) indicated that the identified differences of tinnitus loudness dependency on age and pitch seemed to be similar in both HL groups.

## Discussion

### Hearing Thresholds in Patients with and without Tinnitus

In this retrospective study, we compared audiometric data of a total of more than 37,000 patients (74,976 ears) with sensorineural or CHL with or without tinnitus. We hypothesize that tinnitus is an epiphenomenon of a neuronal process normalizing impaired hearing thresholds. The audiometric results and tinnitus frequency distributions of tinnitus patients of different age groups are in line with the literature [e.g., Ref. (17, 26, 27)] even though the sample groups investigated there were much smaller than in our patient cohort. Also, the hearing thresholds of non-tinnitus patients are comparable to other cohorts (28, 29). Only few studies compared both patient groups directly (30, 31) and found either no change or a worsening of speech perception in tinnitus patients. To our knowledge, no peer reviewed study compared the pure tone hearing thresholds of both patient groups the way we did.

**Figure 7:**
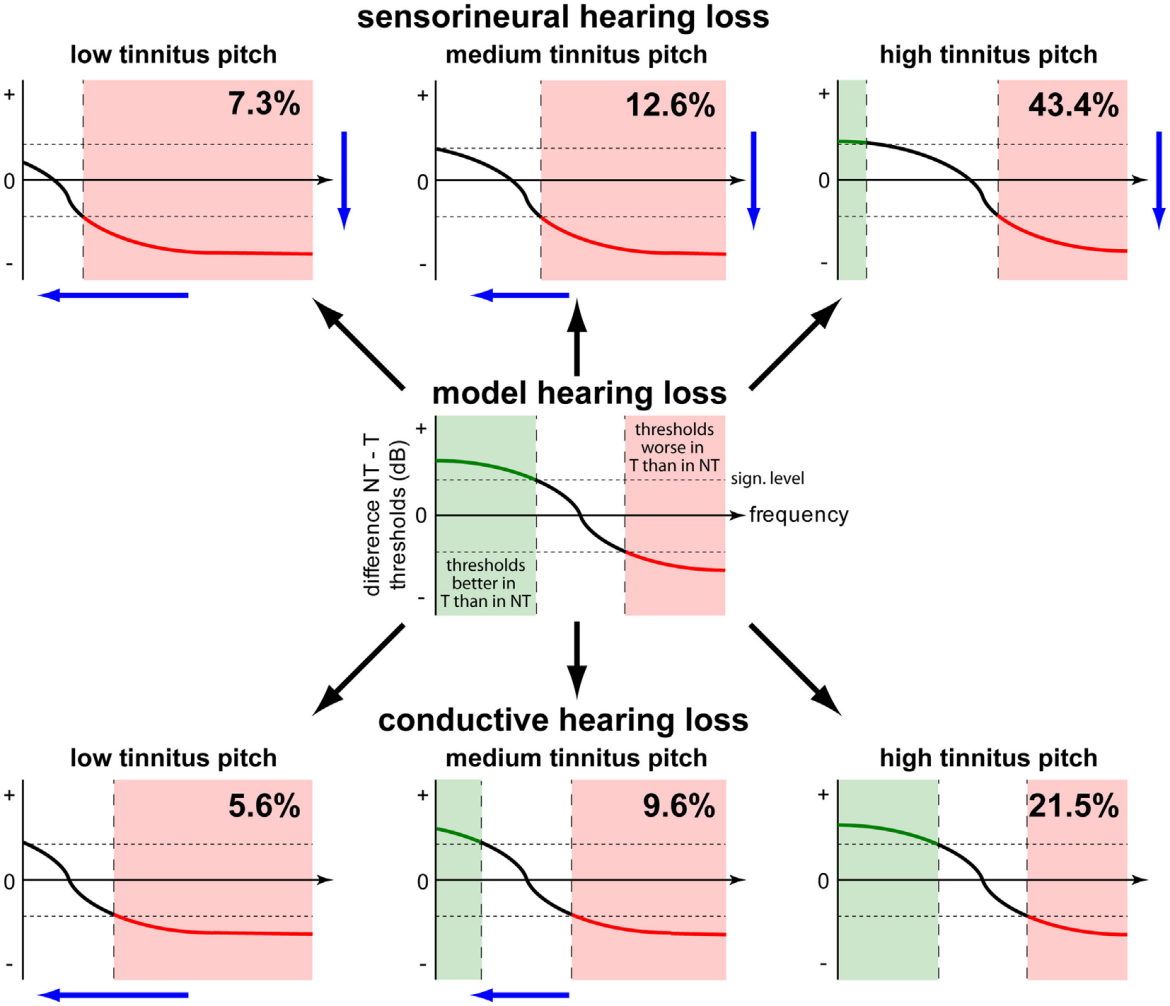
**Model of decreasing (green) or increasing (red) effects of tinnitus on hearing thresholds**. In the center panel, the general effect of tinnitus on hearing threshold is given. Tinnitus is beneficial for thresholds in the low frequency range and detrimental in the high frequency range. The horizontal broken lines symbolize the significance levels. Two main factors alter this relationship by shifting the function either down (SHL) or left (tinnitus pitch). For more explanation, refer to the text.

Here, we report specific differences in hearing thresholds between T and NT patients that may be interpreted as gains or losses in patients with tinnitus compared to non-tinnitus patients. These differences emerged on the population level of over 37,000 patients and in the paired analysis of a smaller subpopulation of roughly 2,500 patients in both pure tone and speech audiometry that seemed to follow a certain systematic (Figure 7, center panel): in T compared to NT adults, lower thresholds were seen in the low frequency range only, while higher thresholds were observed the high frequency range. In addition, lower thresholds were more often seen in CHL patients, while in SHL patients, higher thresholds predominated. Finally, the individually perceived tinnitus pitch seemed to be correlated with systematic alterations to the differences in hearing thresholds (Figure 4), which obviously are more complex than the simple masking we had first hypothesized to explain threshold increasing effects (see Hearing Thresholds in Adults with and without Tinnitus). In the case of paired data (Figure S1 in Supplementary Material), it becomes clear additionally that the most frequent (high) tinnitus pitch leads to the strongest improvement in speech intelligibility. Children and adolescents did not seem to follow this systematic, as only lower thresholds were seen there (Figure 1).

Based on our observations, we here put forward a model of tinnitus impact on hearing thresholds as shown in Figure 7: the basic feature of the model (Figure 7, center panel) is that thresholds in the low frequency range are lowered in tinnitus patients, i.e., in the range where HL is typically mild (Figure 7, center panel, green shaded area). Here, high frequency thresholds are higher in tinnitus patients, i.e., in the range where HL is typically more severe (Figure 7, center panel, red shaded area). This general scheme of hearing threshold change in tinnitus patients may be shifted by two main factors: sensorineural HL can shift the threshold function down (Figure 7, upper row)—CHL (Figure 7, lower row) has no effect on the function, while tinnitus pitch may shift the function along the abscissa (Figure 7, columns), resulting in a larger frequency range of lower thresholds (green shaded areas) for high-pitched tinnitus and a larger frequency range of higher thresholds (red shaded areas) for low-pitched tinnitus.

This model qualitatively reproduces the results of our retrospective data analysis (see Figures 2B,D and 4B,D). Interestingly, when analyzing the relative frequency of the different tinnitus–threshold-interaction types postulated here (percent values in panels of Figure 7), it became obvious that interaction types with predominantly higher hearing thresholds are less frequent (adding up to 25.5%) than those types with lower thresholds (adding up to 74.4%). As these lower thresholds were exclusively observed for pure tone measurements in the low frequency range, we here put forward the hypothesis that *the biological function of the neuronal mechanism that finally also leads to tinnitus is to improve speech perception in the case of hearing loss*. This seems to work particularly well for high-pitched tinnitus which was encountered in about 2/3 of the patients (64.9%). In other words, patients suffering from tinnitus might be affected by this phantom percept as a side effect of their auditory system compensating for HL (cf. below).

### Models of Tinnitus Development

Recent tinnitus models (32–37) all postulate damage to the peripheral receptor epithelium to be etiologic for the development of tinnitus. In response to the decreased input into the auditory system caused by such damage, one class of models further suggest an increased neuronal gain to provide homeostatic plasticity, while other suggest misbalanced lateral inhibition or a failure of a compensatory mechanism as the source of the phantom percept. Both types of models lack explanatory power since the potential biological function of homeostatic plasticity (in terms of information processing) or misbalanced lateral inhibition remains unclear.

To overcome these conceptual problems, we have recently put forward a model for tinnitus-related development of neuronal hyperactivity that is based on SR to restore hearing thresholds after HL (20). SR refers to the phenomenon that weak signals that are sub-threshold for a given sensor still can be detected and transmitted by that sensor if noise is added to the sensor input (21–24). In that way, SR serves to lift signals above a given hearing threshold and most probably is a mechanism that already works in the healthy system (38–40). One could interpret earlier results in a way that SR (while not mentioned explicitly) may also counteract increased hearing thresholds (17, 41). In this interpretation, tinnitus would be a (condoned) side effect of threshold restoration (42). In this context, recent studies have shown that patients with tinnitus show poorer listening performance in noise than patients without tinnitus (30). This is in line with our results, as the researchers focused mainly on sensorineural HL patients with and without tinnitus where we find comparable effects in our large patient cohort.

The analysis of audiometric patient data presented here are perfectly in line with the view of our model: first, the decreasing effects of SR on hearing threshold predicted by our model were observed in all adolescents and child HL patients with tinnitus as well as 65% of the adult HL patients with tinnitus. Second, threshold decreasing effects in tinnitus patients were observed in the low frequency range where HL is mild, that is, in a range where SR should be more effective as the amount of internal noise to be added to the signal to lift it above threshold must not be too high. Third, the threshold decreasing effects were most frequently observed in CHL tinnitus patients and were rather uncommon in SHL patients, which is in line with the idea that an intact neuronal system (as in CHL) more likely is able to compensate for HL by means of a neuronal SR mechanism than a damaged system (as in SHL). Note that still not all CHL patients may show lower thresholds, as patients suffering from CHL and SHL would be classified as CHL based on the diagnostic criteria (mean air-bone gap >5 dB; see Materials and Methods) and, therefore, may reduce the effect in the group. Finally, our model seems biologically plausible in that tinnitus development is condoned as a side effect of threshold restoration, as it particularly improves the speech intelligibility (improved thresholds in the speech relevant frequency range, most frequent (high) tinnitus pitch leads to strongest improvement of speech intelligibility), and communication by speech is crucial for social interaction and communication between humans (2, 25, 43). This view is supported by the fact that high pitch tinnitus patients show the largest distance of speech and corresponding pure tone thresholds, which are even higher than those of NT patients. An alternative interpretation of the data presented here is that steeper audiogram slopes due to better hearing thresholds at low frequencies and worse hearing thresholds at high frequencies are more likely to lead to the development of tinnitus (17).

One drawback of this retrospective study is the lack of information on etiology, comorbidities or medication of the 37,000 patients pooled together here. As tinnitus is only a symptom resulting from different diseases, its specific characteristic may differ from case to case. The fact that our analysis still yielded highly systematic results further strengthens our interpretation of the functional benefit of tinnitus on hearing thresholds.

In conclusion, we think that within an evolutionary frame of reference it makes perfectly sense to optimize hearing and information extraction ability on the cost of generating a phantom percept off the frequencies of interest, i.e., high-pitched tinnitus. Why some patients suffer from low or medium-pitched tinnitus remains unclear and may have different reasons than the threshold decreasing high pitch tinnitus. Another open question why only a minority of CHL patients is developing a tinnitus percept has to be addressed in a more detailed, possibly prospective, follow-up study.

## Author Contributions

DG analyzed the data and wrote a part of the manuscript. KT wrote a part of the manuscript, interpreted the data, and performed the statistical analysis. PK and AS provided assistance in the statistical analysis and interpretation of the data. UH provided the data. HS wrote a part of the manuscript.

## Conflict of Interest Statement

The authors declare that the research was conducted in the absence of any commercial or financial relationships that could be construed as a potential conflict of interest.
