# Supplementary figures and images for: Analysis of Audiometric Differences of Patients with and without Tinnitus in a Large Clinical Database
Source: Front Neurol. 2017 Feb 9;8:31. doi: 10.3389/fneur.2017.00031 (PMC5298966; doi:10.3389/fneur.2017.00031)

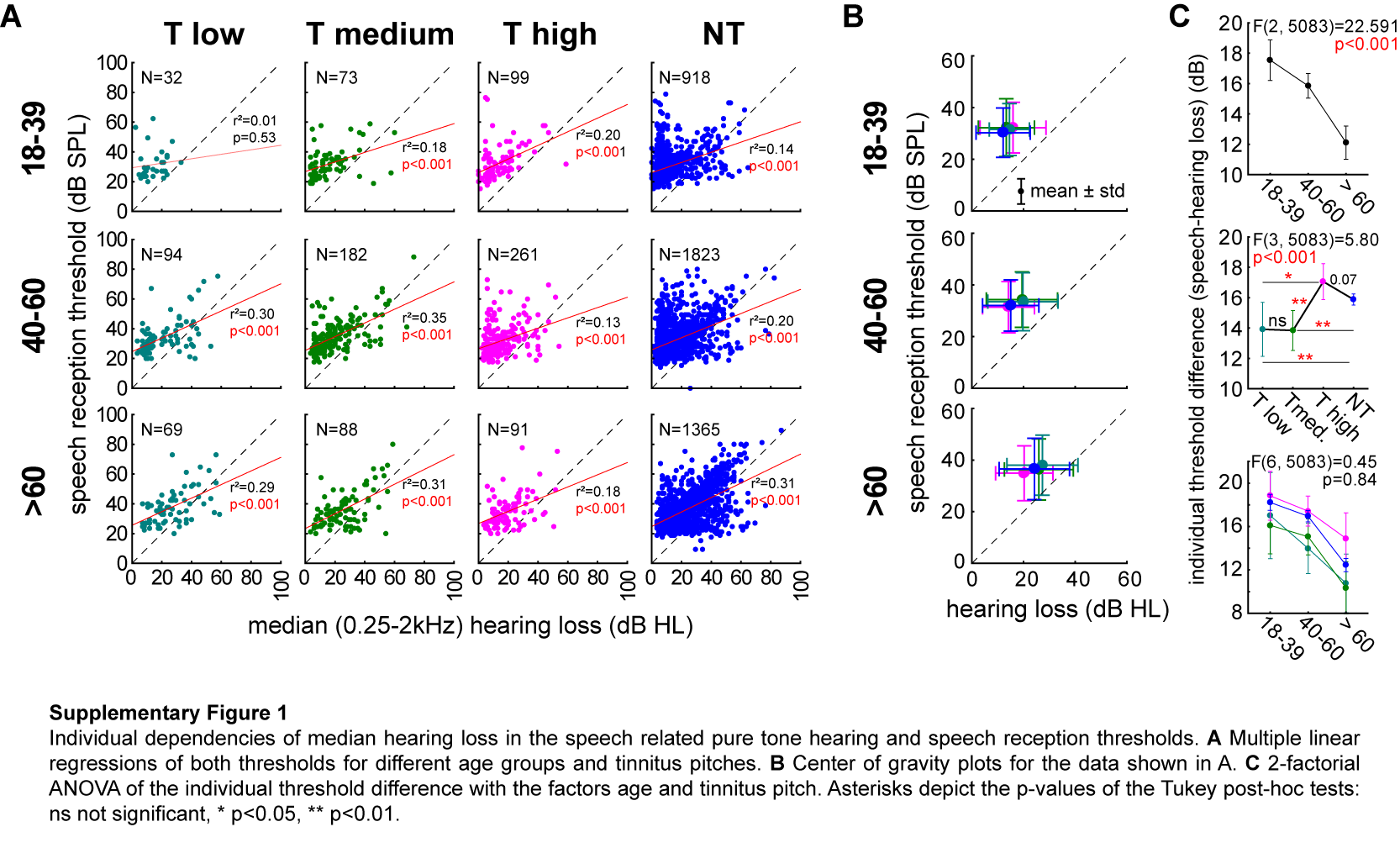

Supplement: Supplementary file 1 [file Image_1.tif]
